# Pore-throat structure characterization of carbon fiber reinforced resin matrix composites: Employing Micro-CT and Avizo technique

**DOI:** 10.1371/journal.pone.0257640

**Published:** 2021-09-22

**Authors:** Yong Li, Yanmeng Chi, Shanling Han, Chaojie Zhao, Yanan Miao

**Affiliations:** 1 College of Mechanical and Electronic Engineering, Shandong University of Science and Technology, Qingdao, China; 2 Sinopec Research Institute of Safety Engineering, Qingdao, China; 3 College of Safety and Environmental Engineering, Shandong University of Science and Technology, Qingdao, China; University of Vigo, SPAIN

## Abstract

Micro-CT technique poses significant applications in characterizing the microstructure of materials. Based on the CT three-dimensional(3D) reconstruction technology and “Avizo” 3D visualization software, the microscopic pore-throat structure of porous media can be quantitatively characterized. This paper takes the carbon fiber reinforced resin matrix composites as an example to introduce the operation process of “Avizo” in details, which mainly covers the following modules: Volume Edit, Interactive Thresholding, Fill Holes, Mask, Separate Objects and Generate Pore Network Model, then further discuss the difficult problems when the “Avizo” is employed to analyze. The microstructures of carbon fiber reinforced resin matrix composites illustrate that pores in the upper part of sample are dramatically dispersed, and mainly concentrated in the lower part of sample. The porosity of adopted cuboid is 3.6%, accordingly the numbers of pores and throats reach 268 and 7, respectively. The equivalent radius of pores seems mainly distributed in the range of 0.7–0.8μm, accounting for 28.73% of the total pore number. The surface area of pore ranges from 5 to 10μm^2^, accounting for 14.16% of the total pore number. The pore volume concentrates in the range of 1–20μm^3^, accounting for 57.46% of the total pore number. In addition, the equivalent radius of throat mainly concentrates in the range of 1–5μm, the overall length of throat is distributed in the range of 37–60μm, and the equivalent area of throat is distributed non-uniformly in the range of 5–75μm^2^. This work provides a basis for the further investigation of fluid migration mechanism and law in the composite materials by the numerical simulation methodology.

## 1 Introduction

As the name implies, the carbon fiber composites stands for the composites with carbon fiber as the reinforcing phase. The matrix of this kind of composites may be resin, metal and ceramic, among which carbon fiber-epoxy resin composites is the mostly common. Carbon fiber reinforced epoxy resin matrix composites belongs to the advanced composite materials, which poses the following excellent properties [1–3]: high specific strength and large specific modulus; Fatigue resistance and damage safety; Thermal conductivity and electrical conductivity. Different from macroscopic defects in metal parts, there are special types of defects in the carbon fiber composites. Generally, the defects formed during the manufacturing process of carbon fiber composites [4] mainly include: porosity, detachment, warpage, delamination, looseness, inclusion, interface separation, cross-layer fracture, poor or rich glue, incorrect resin content, uneven fiber, etc. The existence of above-mentioned defects will bring negative impacts on the properties of composite materials, thus it is necessary to detect the defects in engineering applications, such as porosity, delamination, etc..

Existing studies have gradually demonstrated that pores are commonly caused by the following three reasons: (1) poor infiltration of resin and fiber, air accessed and not excluded during the prepreg preparation or layup; (2) Low molecular components in the solvent used to dilute the resin emit gas during the processing process, or some resin systems emit gas during the curing reaction, such as phenolic resin system, etc.; (3) Improper process control in the preparation process, such as: the relatively small or late pressure will cause the pores be excluded then formed. Abundant of pore characteristics in carbon fiber composites present above micron level, accordingly, the porosity study is of great significance for the flow characterization.

At present, the porosity measurement approaches [5–12] include the mass volume, mercury injection, gas adsorption, metallographic and ultrasonic detection, etc. Mass volume approach is simple to be operated, where the internal pore distribution is unavailable to be known, thus this accuracy seems lower. Mercury injection method is merely suitable to acquire the open porosity, whose results are closely related to the pressure and present the larger error. Gas adsorption methodology is commonly acknowledged as suitable for the measurement of nanoscale pores, however, the lower accuracy is acquired for the medium-low density carbon fiber composites owing to its larger pore sizes or smaller specific surface area. Metallographic measurement results are closely related to the adopted location of sample section, thus the volumetric porosity of sample is hardly reached. The above-mentioned approaches are classified as destructive detection means, accordingly, which is difficult to identify the specific distribution of pores inside the material. Therefore, numerous scholars began to investigate the application of Non-destructive Testing (NDT) for the porosity measurement. A large number of researches on the ultrasonic detection approach of the porosity of composite materials have been conducted [13, 14], unfortunately, which is hardly applicable to the porous carbon fiber composites. In recent years, with the development of microscopic CT technology, the imaging resolution has successfully reached the micron level, which can be employed to analyze the microstructure of composite materials. Zhou et al utilized the X-ray micro-Computed Tomography (X-ray μCT) with a uniaxial compression facility to investigate the evolution of fracture networks in coal during loading [15]. Dewanckele et al performed the high resolution X-ray tomography to visualize gypsum crust formation then yield a better insight into the effects of gaseous SO_2_ on the pore modification in 3D [16]. Notably, “Avizo” software can process the data from X-ray tomography: CT, micro-/nano-CT, electron microscope, and synchrotron, which will precisely calculate the porosity, analyze the pore connectivity and skeleton the pore network modeling for the multi-scale and multi-mode data.

This paper takes carbon fiber reinforced resin matrix composites as an example, introduces the operation process of “Avizo” and discusses the difficult problems when “Avizo” is employed for analysis, which mainly covers the following modules: Volume Edit, Interactive Thresholding, Fill Holes, Mask, Separate Objects, Generate Pore Network Model, as well as Pore Network Model View. The pore structure of carbon fiber composites is quantitatively characterized by both the pore model and ball-bat model. This work provides a basis for further investigation of fluid migration mechanism and law in composite materials by numerical simulation methodology.

## 2 Sample preparation and testing

### 2.1 Experimental sample

The test sample was a piece of cylindrical carbon fiber material with a height of 4.3cm and a diameter of 7cm, which was placed on the scanning table of X-ray three-dimensional microscope. To ensure the accuracy of scanning, the carbon fiber should be fixed as far as possible during the rotation of sample table.

### 2.2 Experimental principle and test process

Non-destructive Testing employs the acoustic, optical, magnetic and electrical characteristics of substances to detect whether the defects and inhomogeneity are existed in the tested objects or not, then further offer information on the size, location, nature and quantity of defects. Industrial CT is a short for industrial computed tomography imaging, which realizes the accurate and clear display on the internal structures, components, materials and defect status of detected objects by employing the two-dimensional (2D) sectional image or 3D-CT image under the non-destructive condition. Therefore, the industrial CT has been treated as the best technique for nondestructive testing and evaluation.

The absorption capacity of material radiation is closely related to material property. By employing the radionuclides or X/γ rays emitted from other radiation sources, the attenuation and distribution characteristics of detected object can be acquired [17, 18]. Accordingly, the computer information processing and image reconstruction technology are utilized to display as the image form.

## 3 Quantitative characterization of the pore model

### 3.1 Image reconstruction

A high resolution X-ray microscope (nano-Voxel-3000) is used to scan the carbon fiber reinforced resin matrix composites at 80kV and 200μA energy levels. The scanning frame number is 1440, the exposure time is 0.5s, and the penetration rate is 80%. The values of SOD and SDD are 236.676mm and 972.192mm, respectively. In each reconstructed image, the resolution of voxel is 2500×2000×2500, and FDK algorithm [19] is employed for 3D image reconstruction. Based on the 3D reconstruction technology, the 3D visualization software “Avizo” is employed to reconstruct the data.

**Fig 1:**
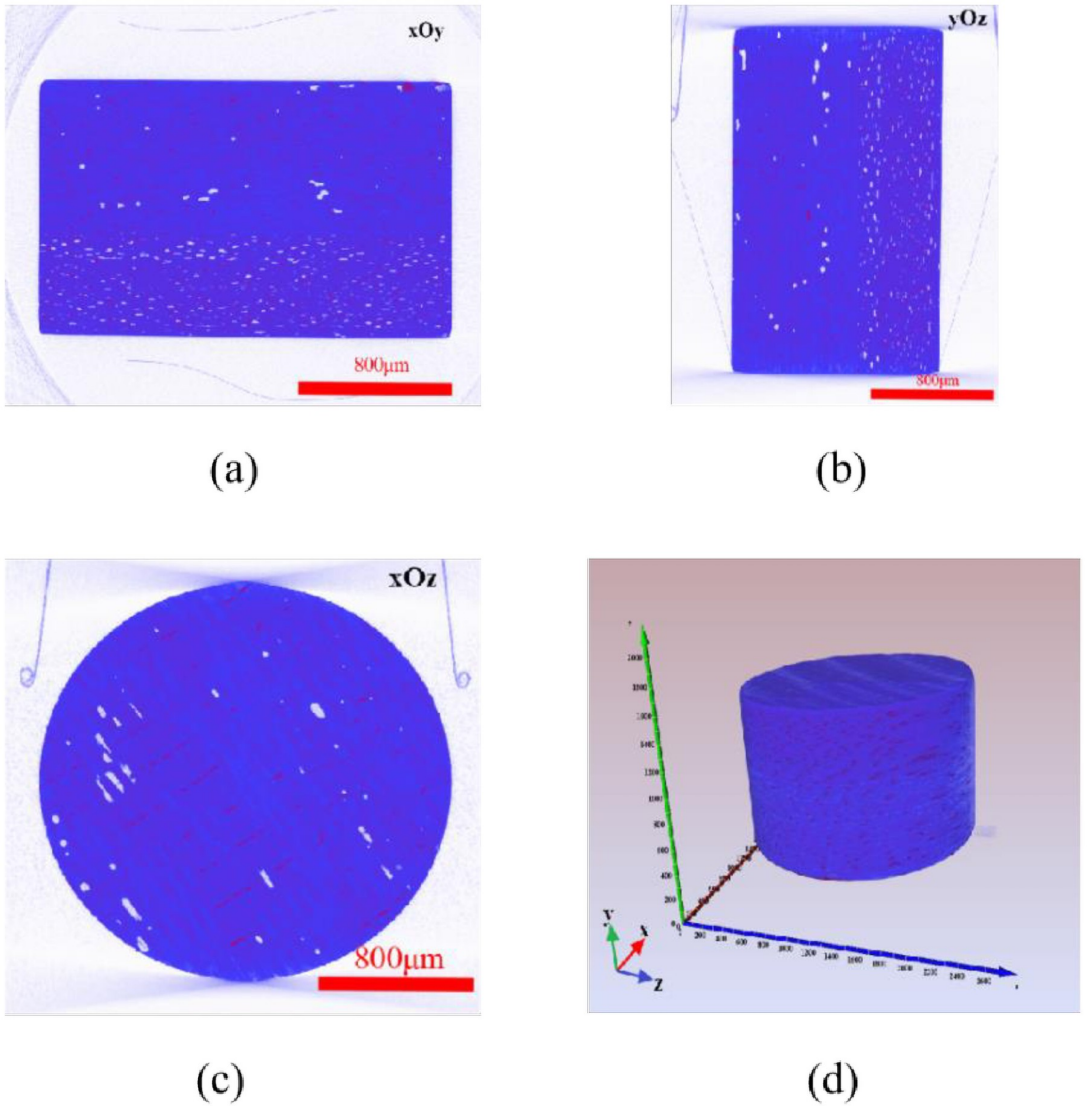
Scanning slices and three-dimensional data in X, Y and Z directions: (a), (b) and (c) are scanned slices in the direction of xOy, yOz and xOz respectively, and (d) are three-dimensional data.

Scanning slices in X, Y and Z directions and 3D data (a cylinder with a radius of 1063.94μm and a height of 1245.15μm) of the composite material are shown in Fig 1. The blue circular area stands for the carbon fiber reinforced resin matrix composites, and the white part inside the material is the void. As can be seen from Fig 1(a), the pores are dispersed in the upper part of cylinder, mainly concentrated in the lower part of cylinder.

### 3.2 Image segmentation

Image segmentation technology is mostly applied into the image early-processing depending on its own characteristics and purposes. Currently, the traditional image segmentation algorithms have been widely utilized as follows [20–22]: threshold-based image segmentation and specific theory-based image segmentation, such as the watershed segmentation algorithm proposed by Vincent et al [23]. In addition, the region-based image segmentation and edge-based image segmentation methods have gradually been introduced.

The earliest algorithm adopted for the image segmentation is threshold processing. Owing to its simple operation and perfect segmentation effect, as well as the sensitive noise through the analysis of gray characteristics, it is commonly utilized for samples presenting the larger discrepancies between the target and background, that is, the bigger contrast. The image segmentation methods are generally divided into two kinds based on the numbers of threshold values, one is determined by a single value for image segmentation, the other is employing multiple thresholds for image segmentation. The ultimate purposes of each approach are acquiring the better segmentation consequences for the subsequent processing [24, 25].

**Fig 2:**
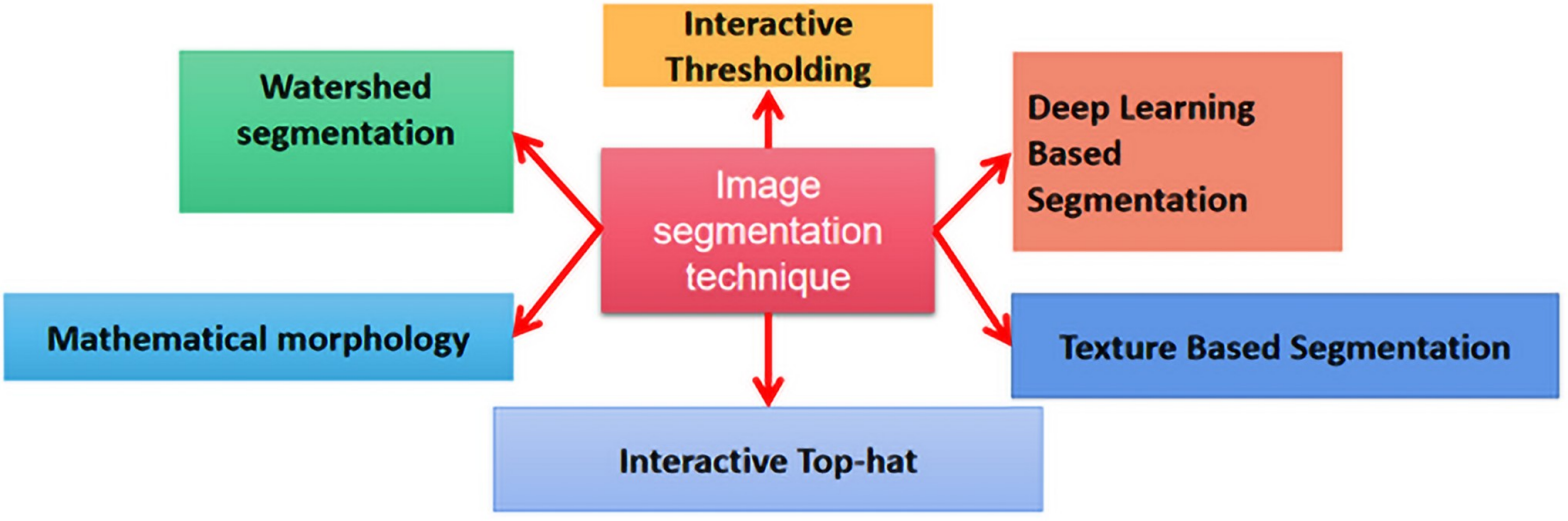
Image segmentation module in “Avizo” software.

The image segmentation module employed commonly in “Avizo” is systematically illustrated in Fig 2. The Interactive Thresholding Module is image segmentation realized by thresholds that will be allowed to select interactively in this module. The current selection is displayed as an overlay on each view of the connected Ortho Views. Press the Apply button to create the binary image. A new field is created that is 1 for each value within the threshold interval and 0 for all other field values. When segmenting more than two phases, a transition between high and low intensity phases may introduce artifacts with unwanted intermediate "coating" phase. Variations in illumination or intensity across image may lead to different thresholds on different regions. The watershed technique provides an effective solution for these issues in many cases. Interactive Top-Hat is a powerful tool for segmenting areas with non-uniform backgrounds, when simple thresholding fails to capture wanted features without unwanted noise. In addition, Image Segmentation methods in “Avizo” are consisted of Mathematical Morphology, Texture Based Segmentation and Deep Learning Based Segmentation.

**Fig 3:**
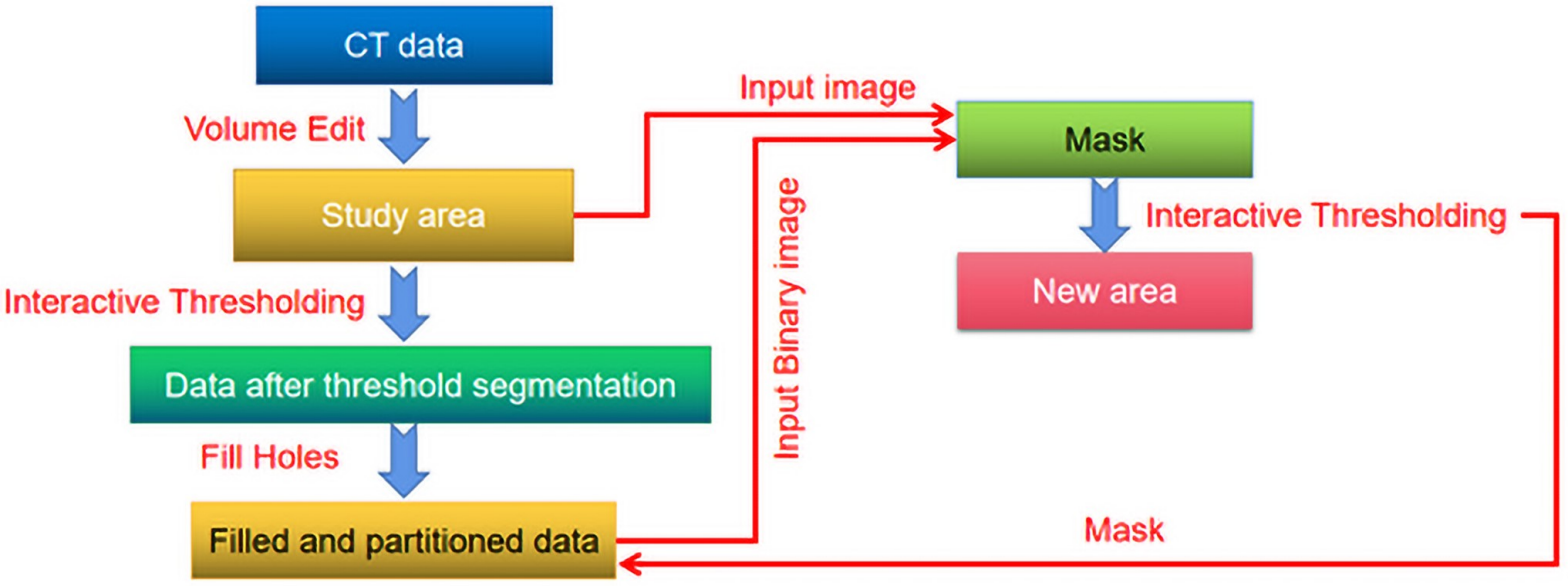
Modular operation flow diagram of pore extraction in carbon fiber composites.

**Fig 4:**
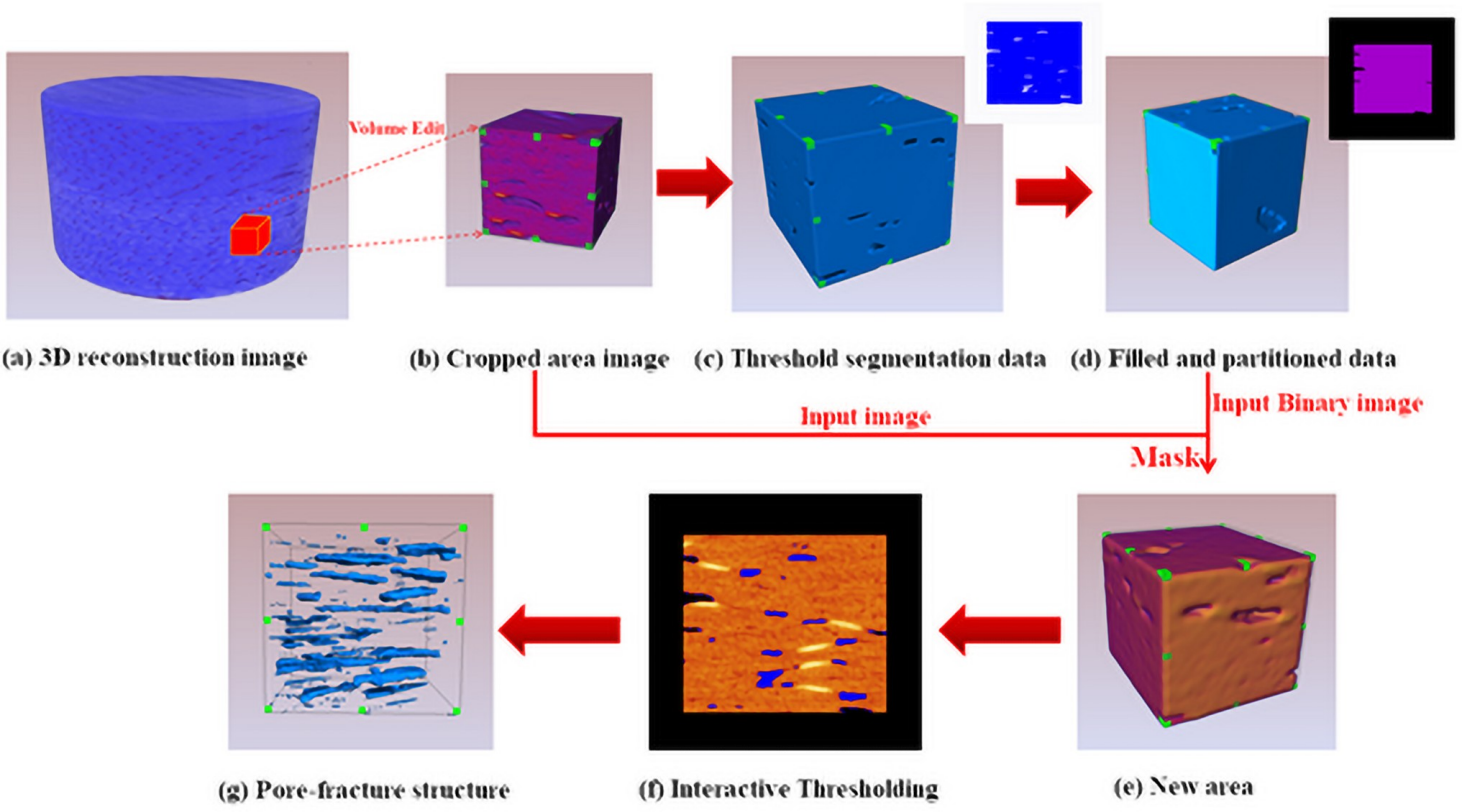
Image segmentation process of pore extraction in carbon fiber composites.

Due to the explicitly clear color of sample slice and background images, the Interactive Thresholding module is employed to extract the pores; however, the gray values between the target and background pixel are relatively close, then the background will be mistakenly selected as pores during the segmentation process. Here, we adopt the Mask module in “Avizo” software to segment the scanned target and background images, and then extract the pore structure in the specific target area. Considering the computing ability and storage capacity of computer, the cuboid area with the length of 165.936μm, width of 152.88μm and height of 144.47μm is selected from the sample as the research object in this work, then the Volume Edit module is employed for operation. In addition, we employ the Interactive Thresholding module and Fill Holes module to segment and fill the inner pores of cuboid, and utilized the Mask module associating the partitioned cuboid images with the filled ones. Accordingly, we utilize the Interactive Thresholding module and associated the Filled and Partitioned Data to extract the pores, which is explicitly illustrated in Fig 3. The image segmentation process of pore extraction in carbon fiber composites is shown in Fig 4. Finally, the pore-throat structure models of carbon fiber reinforced resin matrix composites are obtained, which is sufficiently prepared for the further analysis of ball-bat models. Notably, the Volume Fraction module is utilized for the pore-throat structure model and the cut cuboid area, which presents the 3.6% porosity. Through the further analysis of pore model in carbon fiber composites, the pore radius, pore volume and pore surface area parameters are obtained as shown in Table 1.

**Table 1:** Quantitative parameter statistics of pore model in carbon fiber composites.

Parameters	Pore radius/μm	Pore volume/μm^3^	Pore surface area/μm^2^
Maximum	15.98	17093	8701.87
Average	2.4752	532.053	381.14
Minimum	0.62035	1	3.00419

## 4 Quantitative characterization of the ball-bat model

To investigate the internal pore structure characteristics of samples more precisely, extracting the corresponding pore network model for internal structure analysis is particularly significant. Based on the pore-throat structure model of carbon fiber reinforced resin matrix composites, the ball-bat model is constructed in this work.

The pores are first separated by employing the Separate Object module, whose parameters are indicated in Table 1. Skeleton-Aggressive module labels and separates particles from a Binary Image representing a group of particles. This module generates a Label Field using a separation algorithm watershed based, which carefully sets the seeds at the particles center. The right set procedure of seeds in the particles includes: Calculating the particles distance map; Extracting the particles skeleton (medial axis); Masking the distance map with the skeleton; Finding and labeling the particle center (seeds) on the masked distance map with a H-Maxima module. The number of seeds can be adjusted with the H-Maxima contrast option (calibrating the model), by employing the port Marker Extent. The seeds are then used, together with the inverted distance map, as input to the watershed algorithm to separate the particles [26]. In the Interpretation part, the module configuration is set to 3D. The image will be processed as a whole in 3D. In 3D configuration, neighborhood port refers to the type of connectivity considered for processing adjacent voxels: voxels with at least one common vertex are considered connected. Marker Extent port specifies a number used as a contrast factor to reduce the number of seeds for the watershed. Higher values increase the number of merged seeds, therefore reduce the final number of remaining seeds. It has the same meaning as the contrast factor of H-Maxima. Algorithm Mode port selects the type of algorithm used to separate the objects. It is one of the following said repeatable: repeatable but slower. Output Type port selects the content of the output image. It is one of the following: connected object: Labeled and separated particles.

**Fig 5:**
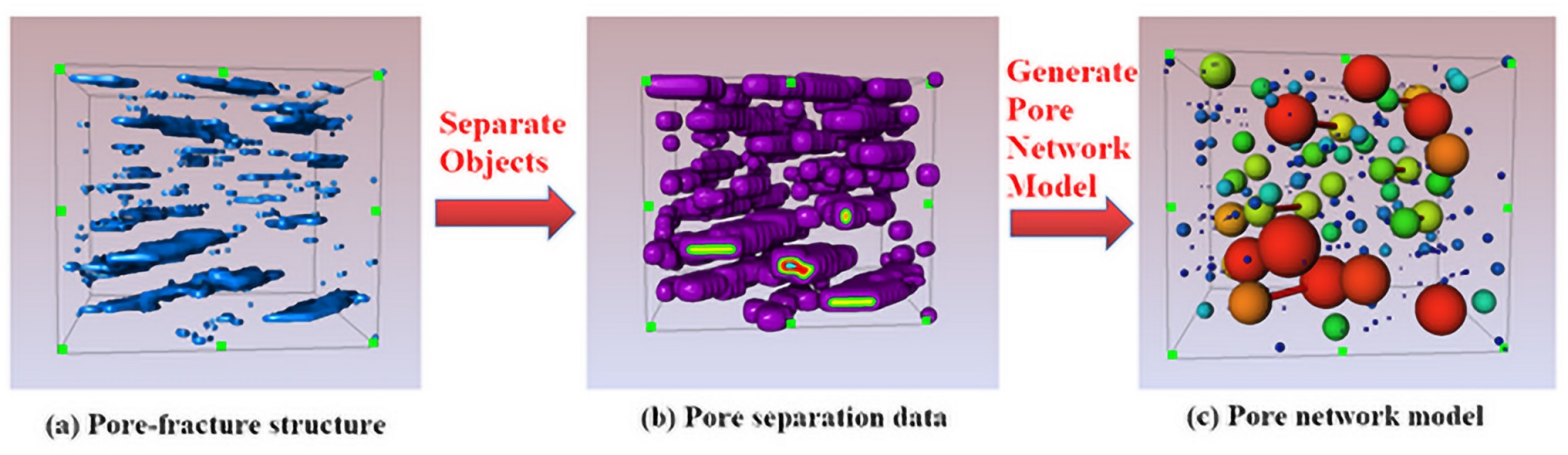
Analysis process from the pore model to ball-bat model.

Generate Pore Network Model module is used to generate the ball-bat model. This module takes one input: a labeled image representing the separated pore space. It outputs the corresponding Pore Network Model. The extracted Pore Network Model contains the following statistics: Number of nodes, number of throats, coordination number throat, equivalent radius, throat channel length (defined as distance from pore to pore centers), throat flow rate per second (if Generate Properties is enabled by the user), pore volume, pore equivalent radius. Use Pore Network Model View to visualize the bat model, and this module is a visualization module for Pore Network Model. Pores are displayed using spheres and throats are presented by cylinders. Each of them may be colored or scaled according to their attributes. Pores and throats can be highlighted by selecting them in the Pore Network Model inner spreadsheet. The size and color of pores can be illustrated in terms of equivalent radius, accordingly, the size and color of throats can be shown in terms of the length of channel. The process generated from the pore model to ball-bat model is presented in Fig 5. The parameters of Separate Object module and Pore Network Model View module are listed in Table 2.

**Table 2:** Parameters utilized in separate objects and generate pore network model modules.

Separate Objects		Pore Network Model	
Method	Skeleton-Aggressive	Pores	
Interpretation	3D	Pore Scale	EqRadius
Neighborhood	26	Pore coloring	EqRadius
Marker Extent	1	Throats	
Output	connected object	Throat Coloring	ChannelLength
Algorithm Mode	repeatable	Throat Scale	ChannelLength

Generate Pore Network Model in “Avizo” is available to generate the pore network model based on the pore structure, and quantitatively characterizes the relevant parameters of pore and throat. It can be reached that the numbers of pores reach 268, and the numbers of throat yield 7. The porosity of adopted cuboid seems 3.6%. The detailed parameters are presented in Table 3.

**Table 3:** Quantitative parameter statistics of pore network model in carbon fiber composites.

Parameters	Pore radius /μm	Pore volume/μm^3^	Pore surface area/μm^2^	Throat radius/μm	Throat length/μm	Throat surface area/μm^2^
Maximum	14.1835	11952	4514.33	4.9060	60.1686	75.614
Average	2.4783	491.9627	352.9295	2.8495	47.6337	31.4331
Minimum	0.6204	1	3.0042	1.3179	37.7293	5.45674

**Fig 6:**
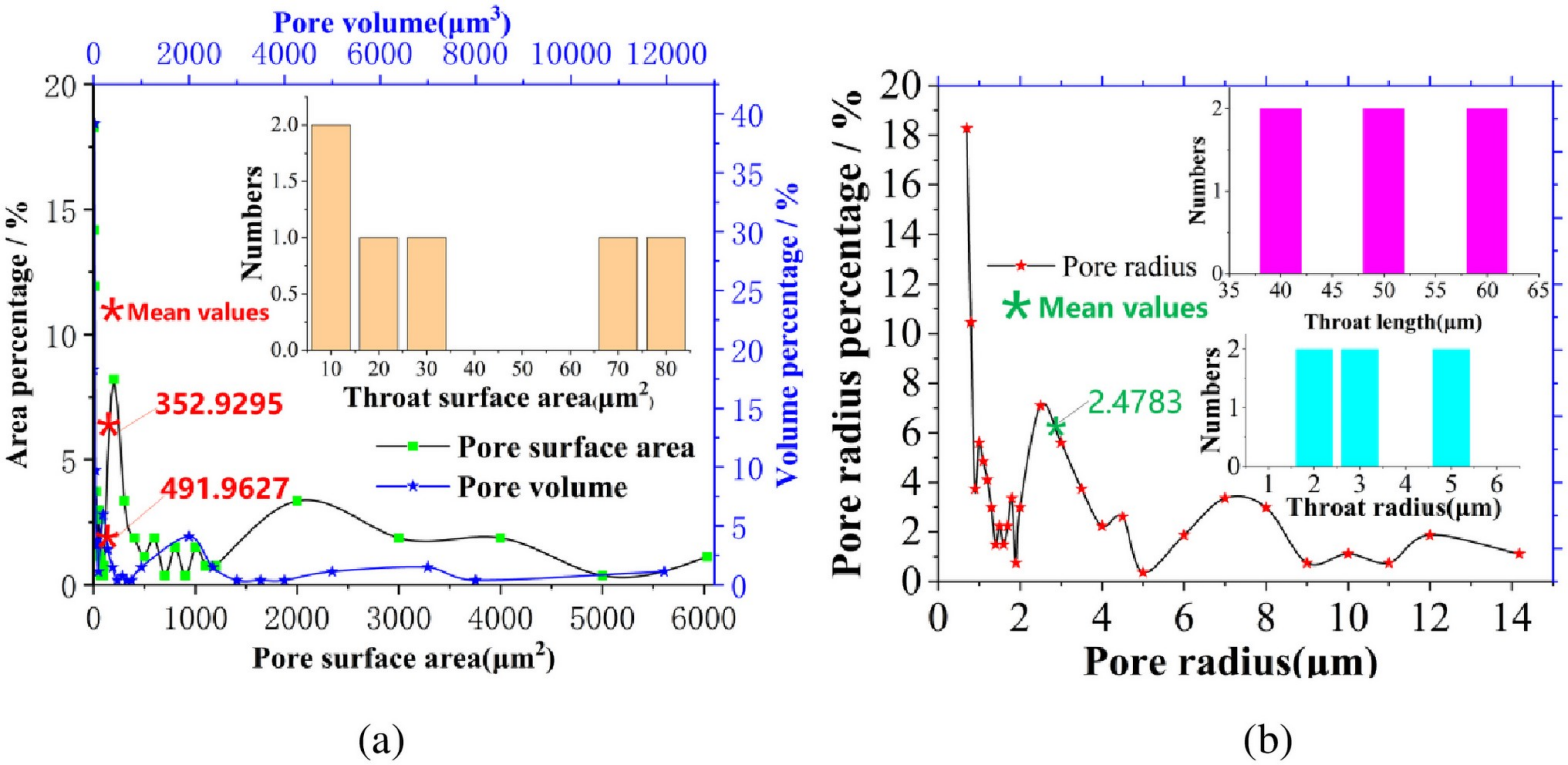
Statistics of pore-throat quantitative parameters in the pore network model.

To further classify the pore structure of carbon fiber reinforced resin matrix composites, the quantitative statistical diagram of the structure in carbon fiber reinforced resin matrix composites is described in Fig 6. The equivalent pore radius of composite material is mainly distributed within the range of 0.7–0.8μm, accounting for 28.73% of the total pore number. The pore surface area is concentrated in the range of 5–10μm^2^, accounting for 14.16% of the total area. The pore volume is mainly distributed in the range of 1–20μm^3^, accounting for 57.46% of the total. It should be noted that the distribution trend of above-mentioned three parameters is roughly the same. In addition, the equivalent radius of throat mainly concentrates in the range of 1–5μm, the overall length of throat ranges from 37 to 60μm, and the equivalent area of throat is distributed non-uniformly in the range of 5–75μm^2^.

## 5 Discussions

During the process of extracting the pore and throat structures of carbon fiber composites, the Image Segmentation module in “Avizo” software should be appropriately adopted depending on its own requirement and material composition. Besides, the process of data preprocessing and post-processing plays a crucial role in the data acquisition of related parameters.

It is seriously necessary to compute the value of representative element volume (REV) during the analysis of reconstructed model. As described in Fan et al.’s study [17], the main idea to determine the size of REV is to calculate the porosity region of interest (ROI), which takes the corresponding size where the porosity no longer changes as the REV of whole sample. When the porosity of each ROI does not fluctuate significantly, the determined REV size is 350×350×350 voxels. Different from Fan et al.’s study, we adopted a small part of cuboid area in this samplefor the analysis owing to the consideration of computer storage and computing capacity, which illustrates that the length of 165.936μm, width of 152.88μm and height of 144.47μm is selected by this approach.

In addition, we analyzed the pore and throat structures of carbon fiber composite materials by employing the Pore Network model. It should be noted that the pore extraction at the edge of the sample may be insufficient with this methodology, which is also related to the local cuboid area in the selection due to the possibility of pores divided at the edges of the cuboid.

To further investigate the mechanism and law of fluid migration in composite materials by using numerical simulation method, more representative structural parameters can be obtained by selecting a larger area of this sample. Remarkably, the parameter selection of each module in “Avizo” software poses significant impacts on the investigation of fluid migration mechanism and law of material; therefore, the parameters of each module should be appropriately determined depending on the actual analysis conditions.

## 6 Conclusions

(1) Based on the CT three-dimensional(3D) reconstruction technology and “Avizo” 3D visualization software, the microscopic pore-throat structures of porous media are available to be quantitatively characterized.
(2) Taking the carbon fiber reinforced resin matrix composites as an example, we introduce the “Avizo” operation process in detail, which mainly covers the following modules: Volume Edit, Interactive Thresholding, Fill Holes, Mask, Separate Objects and Generate Pore Network model, then further discuss the difficult problems when employing “Avizo” to analyze.
(3) Based on the cuboid region intercepted from this carbon fiber reinforced resin matrix composites sample, the pores in the upper part of sample are dispersed, mainly concentrated in the lower part of sample. Moreover, more irregular pores and less round/elliptical pores are existed in this sample. Notably, the porosity of cuboid region intercepted from this carbon fiber reinforced resin matrix composites sample is 3.6%, the numbers of pores reach 268, and the numbers of throats yield 6.
(4) The equivalent pore radius is mainly distributed in the range of 0.7–0.8μm, accounting for 28.73% of the total pore number; the pore surface area is concentrated in 5–10μm^2^, accounting for 14.16% of the total; the pore volume is mainly distributed in the range of 1–20μm^3^, accounting for 57.46% of the total. In addition, the equivalent radius of throat mainly concentrates in the range of 1–5μm, the overall length of throat is distributed in the range of 37–60μm, and the equivalent area of throat is distributed non-uniformly in the range of 5–75μm^2^.

## Supporting information

S1 Appendix (DOCX)
