# Supplementary material for: Pore-throat structure characterization of carbon fiber reinforced resin matrix composites: Employing Micro-CT and Avizo technique
Source: PLoS One. 2021 Sep 22;16(9):e0257640. doi: 10.1371/journal.pone.0257640 (PMC8457498; doi:10.1371/journal.pone.0257640)
Supplement: S1 Appendix — (DOCX) [file pone.0257640.s001.docx]

**S1 Appendix**

**Pore-throat structure characterization of carbon fiber reinforced resin matrix composites: Employing Micro-CT and Avizo technique**

Yong Li ^a^, Yanmeng Chi ^a^, Shanling Han ^a^, Chaojie Zhao ^b^, Yanan Miao ^c,^*

^a^ College of Mechanical and Electronic Engineering, Shandong University of Science and Technology, Qingdao, 266590, China;

^b^ Sinopec Research Institute of Safety Engineering, Qingdao 266000, China;

^c^ College of Safety and Environmental Engineering, Shandong University of Science and Technology, Qingdao, 266590, China.

*Corresponding author: Yanan Miao, email: miao_yanan@sdust.edu.cn, [tel number: 8](tel:+8)615092207869, fax number: 01089734193.

**CONTENTS**

[Ⅰ. SI Text 1](#_Toc80726021)

[A:Label Analysis 1](#_Toc80726022)

[B:Pore Network Model 1](#_Toc80726023)

[Ⅱ.SI Figures 2](#_Toc80726024)

[Ⅲ.SI Tables 3](#_Toc80726025)

# Ⅰ. SI Text

## A:Label Analysis

This data type represents a read only spreadsheet. As with any spreadsheet, you can display it in the Tables panel, compute its histogram, plot it, query it from Tcl scripts, and import it into an external spreadsheet program like Microsoft Excel.
 When Label Analysis is displayed, two spreadsheets appear in the same table. The first one is a summary of the second one. It is composed of statistics computed on each column (Means, Min, Max, Median, Variance, Kurtosis and Skewness). Label Analysis are generated by [Label Analysis module](../hxquant2custom/HxAnalyzeLabels.html#A1).

## B:Pore Network Model

A Pore Network Model data type is designed to store data that can be represented as linear lines in 3D space and that may be organized in networks of multiple such lines. This data type is a specialization of Spatial Graph. Branching or endpoints of the network are called “Pores”, the lines connecting pores are called “Throats”.

For each pore and throat, one or more scalar data items can be stored. These data are displayed in the spreadsheet which is accessible using the Table port. Instead of displaying three tables like Spatial Graph, there are only two tables: one for the Pores and one for the Throats. The Pores table is composed of the pores ID, the pores attributes, and their 3D coordinates. The Throats table is composed of the throats ID, the throats attributes, the throats source ID, and the throats target ID. See Spatial Graph for more information.

In order to visualize the network of a Pore Network Model object, the module Pore Network Model View can be used. As Spatial Graph objects, Pore Network Model objects can be stored using the Avizo file format. They can also be imported from a set of files of Pore Network Node-Link file format.

This module takes one input: a labeled image representing the separated pore space. It outputs the corresponding Pore Network Model. The extracted Pore Network Model contains the following statistics:

Number of nodes

Number of throats

Coordination number

Throat equivalent radius

Throat channel length (defined as distance from pore to pore centers)

Pore volume

Pore equivalent radius

# Ⅱ.SI Figures


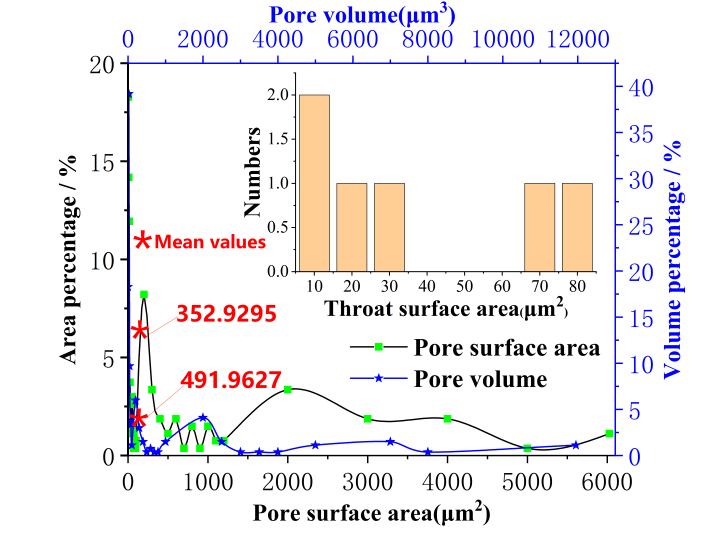

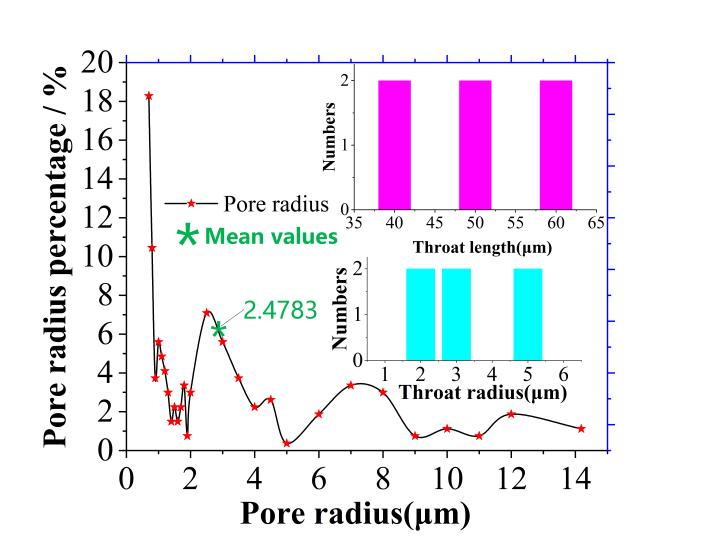


1. (b)

**Fig.S Statistics of pore-throat quantitative parameters in the pore network model**

The data presented in Fig.S(a) is acquired from the pore network model generated by “Avizo” software. This figure shows the pore surface area, the percentages of total pores in the range of 3-6027.34μm and 1-11592μm, the number of pores in the range of 10-80μm, and pore surface area. The pore surface area and pore volume are represented by black and blue lines, and the pore surface area is represented by the histogram in the upper right corner. In this figure, the red * represents the pore surface area and the average pore volume, respectively.

The data in Fig.S(b) is also analyzed and obtained from the pore network model data. This figure shows the percentage of pore number distributing in the range of 0.62-14.18μm. The figure also shows the pore number in the range of 1.32-4.41μm and 37.73-60.17μm, respectively. The green * marks the average pore radius of 2.4783μm.

# Ⅲ.SI Tables

**Table S1 Quantitative parameter statistics of pore model in carbon fiber composites**

| Parameters | Pore radius/μm | Pore volume/μm^3^ | Pore surface area/μm^2^ |
| --- | --- | --- | --- |
| Maximum | 15.98 | 17093 | 8701.87 |
| Average | 2.4752 | 532.053 | 381.14 |
| Minimum | 0.62035 | 1 | 3.00419 |

The data in Table S1 are obtained by Label Analysis module in “Avizo” software, which employs the data statistics methodology.

**Table S2 Quantitative parameter statistics of pore network model in carbon fiber composites**

| Parameters | Pore radius /μm | Pore volume/μm^3^ | Pore surface area/μm^2^ | Throat radius/μm | Throat length/μm | Throat surface area/μm^2^ |
| --- | --- | --- | --- | --- | --- | --- |
| Maximum | 14.1835 | 11952 | 4514.33 | 4.9060 | 60.1686 | 75.614 |
| Average | 2.4783 | 491.9627 | 352.9295 | 2.8495 | 47.6337 | 31.4331 |
| Minimum | 0.6204 | 1 | 3.0042 | 1.3179 | 37.7293 | 5.45674 |

The data in Table S2 is obtained from the pore network model in “Avizo” software, which is analyzed by using the data statistical approach.

**Table S3 Quantitative parameter statistics of pore network model in carbon fiber composites**

| Pore radius /μm | Pore volume/μm^3^ | Pore surface area/μm^2^ | Throat radius/μm | Throat length/μm | Throat surface area/μm^2^ |
| --- | --- | --- | --- | --- | --- |
| 14.1835 | 11952 | 4514.33 | 4.4123 | 60.1686 | 61.1623 |
| 14.0542 | 11628 | 6027.34 | 1.5363 | 44.4109 | 7.41483 |
| 14.0421 | 11598 | 5266 | 2.8319 | 39.4987 | 25.1948 |
| 11.9999 | 7238 | 3982.23 | 2.0925 | 48.5032 | 13.7557 |
| 11.774 | 6837 | 3820.99 | 4.9060 | 55.4917 | 75.614 |
| 11.6651 | 6649 | 3614.7 | 1.3179 | 37.7293 | 5.45674 |
| 11.5844 | 6512 | 5993.43 | - | - | - |
| 11.2906 | 6029 | 2668.66 | - | - | - |
| 10.479 | 4820 | 2986.77 | - | - | - |
| 10.4141 | 4731 | 2658.32 | - | - | - |
| 9.96183 | 4141 | 3132.73 | - | - | - |
| 9.67712 | 3796 | 3064.39 | - | - | - |
| 9.3519 | 3426 | 2334.63 | - | - | - |
| 8.76747 | 2823 | 1604.63 | - | - | - |
| 8.04985 | 2185 | 1485.77 | - | - | - |
| 7.99918 | 2144 | 1468.7 | - | - | - |
| 7.98548 | 2133 | 1527.2 | - | - | - |
| 7.84965 | 2026 | 1524.52 | - | - | - |
| 7.79896 | 1987 | 1498.22 | - | - | - |
| 7.67803 | 1896 | 2156.04 | - | - | - |
| 7.64277 | 1870 | 1940.68 | - | - | - |
| 7.13735 | 1523 | 1748.17 | - | - | - |
| 7.13579 | 1522 | 1180.62 | - | - | - |
| 6.98412 | 1427 | 1015.04 | - | - | - |
| 6.78432 | 1308 | 979.741 | - | - | - |
| 6.68431 | 1251 | 907.957 | - | - | - |
| 6.63225 | 1222 | 1088.56 | - | - | - |
| 6.35487 | 1075 | 1776.57 | - | - | - |
| 6.345 | 1070 | 768.556 | - | - | - |
| 6.18692 | 992 | 962.696 | - | - | - |
| 6.07471 | 939 | 918.932 | - | - | - |
| 6.03346 | 920 | 1172.92 | - | - | - |
| 5.99607 | 903 | 853.287 | - | - | - |
| 5.55493 | 718 | 586.975 | - | - | - |
| 5.35436 | 643 | 786.231 | - | - | - |
| 5.11024 | 559 | 747.863 | - | - | - |
| 5.01713 | 529 | 531.207 | - | - | - |
| 4.67868 | 429 | 619.857 | - | - | - |
| 4.38425 | 353 | 776.164 | - | - | - |
| 4.37596 | 351 | 442.371 | - | - | - |
| 4.29553 | 332 | 502.092 | - | - | - |
| 4.29121 | 331 | 575.068 | - | - | - |
| 4.12028 | 293 | 408.965 | - | - | - |
| 4.08243 | 285 | 464.975 | - | - | - |
| 4.01939 | 272 | 364.085 | - | - | - |
| 3.96952 | 262 | 579.22 | - | - | - |
| 3.81183 | 232 | 293.534 | - | - | - |
| 3.7731 | 225 | 335.525 | - | - | - |
| 3.66967 | 207 | 359.904 | - | - | - |
| 3.63387 | 201 | 243.233 | - | - | - |
| 3.56006 | 189 | 347.013 | - | - | - |
| 3.47649 | 176 | 303.732 | - | - | - |
| 3.43653 | 170 | 194.566 | - | - | - |
| 3.32515 | 154 | 290.911 | - | - | - |
| 3.30341 | 151 | 254.272 | - | - | - |
| 3.30341 | 151 | 255.4 | - | - | - |
| 3.2961 | 150 | 183.859 | - | - | - |
| 3.20575 | 138 | 172.936 | - | - | - |
| 3.11821 | 127 | 217.369 | - | - | - |
| 3.11 | 126 | 196.158 | - | - | - |
| 3.06831 | 121 | 209.353 | - | - | - |
| 2.99914 | 113 | 251.867 | - | - | - |
| 2.95424 | 108 | 147.897 | - | - | - |
| 2.95424 | 108 | 131.139 | - | - | - |
| 2.93589 | 106 | 180.58 | - | - | - |
| 2.89848 | 102 | 190.911 | - | - | - |
| 2.85033 | 97 | 208.06 | - | - | - |
| 2.81059 | 93 | 162.612 | - | - | - |
| 2.6841 | 81 | 142.744 | - | - | - |
| 2.67301 | 80 | 113.83 | - | - | - |
| 2.65055 | 78 | 122.448 | - | - | - |
| 2.60444 | 74 | 129.531 | - | - | - |
| 2.60444 | 74 | 163.973 | - | - | - |
| 2.59266 | 73 | 150.989 | - | - | - |
| 2.55664 | 70 | 141.477 | - | - | - |
| 2.54441 | 69 | 146.873 | - | - | - |
| 2.46841 | 63 | 106.703 | - | - | - |
| 2.44201 | 61 | 139.632 | - | - | - |
| 2.42859 | 60 | 94.6669 | - | - | - |
| 2.42859 | 60 | 99.5039 | - | - | - |
| 2.37338 | 56 | 126.614 | - | - | - |
| 2.30053 | 51 | 100.315 | - | - | - |
| 2.27005 | 49 | 82.5933 | - | - | - |
| 2.2545 | 48 | 102.202 | - | - | - |
| 2.23874 | 47 | 98.429 | - | - | - |
| 2.23874 | 47 | 62.6354 | - | - | - |
| 2.20652 | 45 | 86.6541 | - | - | - |
| 2.17333 | 43 | 61.3307 | - | - | - |
| 2.10374 | 39 | 66.5956 | - | - | - |
| 2.04835 | 36 | 56.9554 | - | - | - |
| 2.04835 | 36 | 85.0345 | - | - | - |
| 2.02921 | 35 | 55.4218 | - | - | - |
| 2.00969 | 34 | 50.4817 | - | - | - |
| 2.00969 | 34 | 50.9049 | - | - | - |
| 2.00969 | 34 | 74.9334 | - | - | - |
| 1.9898 | 33 | 51.8077 | - | - | - |
| 1.94876 | 31 | 77.6144 | - | - | - |
| 1.92757 | 30 | 51.3958 | - | - | - |
| 1.90591 | 29 | 61.366 | - | - | - |
| 1.90591 | 29 | 70.327 | - | - | - |
| 1.90591 | 29 | 48.2225 | - | - | - |
| 1.90591 | 29 | 47.6866 | - | - | - |
| 1.90591 | 29 | 74.7619 | - | - | - |
| 1.83779 | 26 | 48.5361 | - | - | - |
| 1.83779 | 26 | 51.3102 | - | - | - |
| 1.7894 | 24 | 49.3795 | - | - | - |
| 1.7894 | 24 | 58.479 | - | - | - |
| 1.7894 | 24 | 47.2698 | - | - | - |
| 1.7894 | 24 | 40.5647 | - | - | - |
| 1.76419 | 23 | 47.8086 | - | - | - |
| 1.76419 | 23 | 54.6709 | - | - | - |
| 1.76419 | 23 | 54.46 | - | - | - |
| 1.7115 | 21 | 37.1822 | - | - | - |
| 1.7115 | 21 | 41.5927 | - | - | - |
| 1.68389 | 20 | 36.0768 | - | - | - |
| 1.68389 | 20 | 34.027 | - | - | - |
| 1.65534 | 19 | 46.8253 | - | - | - |
| 1.65534 | 19 | 42.5422 | - | - | - |
| 1.65534 | 19 | 48.1236 | - | - | - |
| 1.62578 | 18 | 32.5335 | - | - | - |
| 1.5951 | 17 | 41.6126 | - | - | - |
| 1.56319 | 16 | 30.5171 | - | - | - |
| 1.56319 | 16 | 39.7439 | - | - | - |
| 1.52992 | 15 | 35.3006 | - | - | - |
| 1.49513 | 14 | 32.0278 | - | - | - |
| 1.49513 | 14 | 28.4542 | - | - | - |
| 1.45865 | 13 | 29.3455 | - | - | - |
| 1.42025 | 12 | 25.6543 | - | - | - |
| 1.42025 | 12 | 23.7653 | - | - | - |
| 1.42025 | 12 | 27.7591 | - | - | - |
| 1.37965 | 11 | 24.7549 | - | - | - |
| 1.3365 | 10 | 20.9589 | - | - | - |
| 1.3365 | 10 | 20.3828 | - | - | - |
| 1.3365 | 10 | 24.9059 | - | - | - |
| 1.29038 | 9 | 22.9346 | - | - | - |
| 1.29038 | 9 | 22.2735 | - | - | - |
| 1.29038 | 9 | 21.704 | - | - | - |
| 1.29038 | 9 | 22.5228 | - | - | - |
| 1.29038 | 9 | 22.6403 | - | - | - |
| 1.29038 | 9 | 19.266 | - | - | - |
| 1.2407 | 8 | 19.3094 | - | - | - |
| 1.2407 | 8 | 18.3215 | - | - | - |
| 1.18669 | 7 | 18.3633 | - | - | - |
| 1.18669 | 7 | 15.3624 | - | - | - |
| 1.18669 | 7 | 15.3624 | - | - | - |
| 1.12725 | 6 | 14.6172 | - | - | - |
| 1.12725 | 6 | 14.4097 | - | - | - |
| 1.12725 | 6 | 14.0379 | - | - | - |
| 1.12725 | 6 | 15.9352 | - | - | - |
| 1.12725 | 6 | 16.8214 | - | - | - |
| 1.12725 | 6 | 15.9704 | - | - | - |
| 1.12725 | 6 | 14.5206 | - | - | - |
| 1.12725 | 6 | 15.9851 | - | - | - |
| 1.06078 | 5 | 13.2944 | - | - | - |
| 1.06078 | 5 | 12.5625 | - | - | - |
| 1.06078 | 5 | 12.1891 | - | - | - |
| 1.06078 | 5 | 13.8639 | - | - | - |
| 1.06078 | 5 | 13.5604 | - | - | - |
| 1.06078 | 5 | 12.1907 | - | - | - |
| 1.06078 | 5 | 13.7146 | - | - | - |
| 1.06078 | 5 | 12.5642 | - | - | - |
| 1.06078 | 5 | 12.9293 | - | - | - |
| 1.06078 | 5 | 12.1907 | - | - | - |
| 1.06078 | 5 | 13.2961 | - | - | - |
| 1.06078 | 5 | 12.5625 | - | - | - |
| 1.06078 | 5 | 12.5625 | - | - | - |
| 0.984745 | 4 | 10.1343 | - | - | - |
| 0.984745 | 4 | 10.5012 | - | - | - |
| 0.984745 | 4 | 10.8663 | - | - | - |
| 0.984745 | 4 | 10.5012 | - | - | - |
| 0.984745 | 4 | 10.8663 | - | - | - |
| 0.984745 | 4 | 10.5012 | - | - | - |
| 0.984745 | 4 | 11.2332 | - | - | - |
| 0.984745 | 4 | 10.4995 | - | - | - |
| 0.984745 | 4 | 10.4995 | - | - | - |
| 0.984745 | 4 | 10.7104 | - | - | - |
| 0.984745 | 4 | 10.131 | - | - | - |
| 0.984745 | 4 | 10.1343 | - | - | - |
| 0.984745 | 4 | 10.2903 | - | - | - |
| 0.984745 | 4 | 10.1343 | - | - | - |
| 0.984745 | 4 | 10.131 | - | - | - |
| 0.8947 | 3 | 8.07135 | - | - | - |
| 0.8947 | 3 | 8.07135 | - | - | - |
| 0.8947 | 3 | 8.07135 | - | - | - |
| 0.8947 | 3 | 8.07135 | - | - | - |
| 0.8947 | 3 | 8.07135 | - | - | - |
| 0.8947 | 3 | 8.4365 | - | - | - |
| 0.8947 | 3 | 8.07135 | - | - | - |
| 0.8947 | 3 | 8.07135 | - | - | - |
| 0.8947 | 3 | 8.07135 | - | - | - |
| 0.8947 | 3 | 8.07135 | - | - | - |
| 0.781593 | 2 | 5.64153 | - | - | - |
| 0.781593 | 2 | 5.79915 | - | - | - |
| 0.781593 | 2 | 5.64153 | - | - | - |
| 0.781593 | 2 | 5.64153 | - | - | - |
| 0.781593 | 2 | 5.64323 | - | - | - |
| 0.781593 | 2 | 5.64323 | - | - | - |
| 0.781593 | 2 | 5.64323 | - | - | - |
| 0.781593 | 2 | 5.64323 | - | - | - |
| 0.781593 | 2 | 5.64153 | - | - | - |
| 0.781593 | 2 | 5.79915 | - | - | - |
| 0.781593 | 2 | 5.79915 | - | - | - |
| 0.781593 | 2 | 5.64323 | - | - | - |
| 0.781593 | 2 | 5.79915 | - | - | - |
| 0.781593 | 2 | 5.64323 | - | - | - |
| 0.781593 | 2 | 5.79915 | - | - | - |
| 0.781593 | 2 | 5.64153 | - | - | - |
| 0.781593 | 2 | 5.64323 | - | - | - |
| 0.781593 | 2 | 5.64323 | - | - | - |
| 0.781593 | 2 | 5.79915 | - | - | - |
| 0.781593 | 2 | 5.64323 | - | - | - |
| 0.781593 | 2 | 5.64323 | - | - | - |
| 0.781593 | 2 | 5.64153 | - | - | - |
| 0.781593 | 2 | 5.79915 | - | - | - |
| 0.781593 | 2 | 5.79915 | - | - | - |
| 0.781593 | 2 | 5.64323 | - | - | - |
| 0.781593 | 2 | 5.64323 | - | - | - |
| 0.781593 | 2 | 5.79915 | - | - | - |
| 0.781593 | 2 | 5.64153 | - | - | - |
| 0.62035 | 1 | 3.00419 | - | - | - |
| 0.62035 | 1 | 3.00419 | - | - | - |
| 0.62035 | 1 | 3.00419 | - | - | - |
| 0.62035 | 1 | 3.00419 | - | - | - |
| 0.62035 | 1 | 3.00419 | - | - | - |
| 0.62035 | 1 | 3.00419 | - | - | - |
| 0.62035 | 1 | 3.00419 | - | - | - |
| 0.62035 | 1 | 3.00419 | - | - | - |
| 0.62035 | 1 | 3.00419 | - | - | - |
| 0.62035 | 1 | 3.00419 | - | - | - |
| 0.62035 | 1 | 3.00419 | - | - | - |
| 0.62035 | 1 | 3.00419 | - | - | - |
| 0.62035 | 1 | 3.00419 | - | - | - |
| 0.62035 | 1 | 3.00419 | - | - | - |
| 0.62035 | 1 | 3.00419 | - | - | - |
| 0.62035 | 1 | 3.00419 | - | - | - |
| 0.62035 | 1 | 3.00419 | - | - | - |
| 0.62035 | 1 | 3.00419 | - | - | - |
| 0.62035 | 1 | 3.00419 | - | - | - |
| 0.62035 | 1 | 3.00419 | - | - | - |
| 0.62035 | 1 | 3.00419 | - | - | - |
| 0.62035 | 1 | 3.00419 | - | - | - |
| 0.62035 | 1 | 3.00419 | - | - | - |
| 0.62035 | 1 | 3.00419 | - | - | - |
| 0.62035 | 1 | 3.00419 | - | - | - |
| 0.62035 | 1 | 3.00419 | - | - | - |
| 0.62035 | 1 | 3.00419 | - | - | - |
| 0.62035 | 1 | 3.00419 | - | - | - |
| 0.62035 | 1 | 3.00419 | - | - | - |
| 0.62035 | 1 | 3.00419 | - | - | - |
| 0.62035 | 1 | 3.00419 | - | - | - |
| 0.62035 | 1 | 3.00419 | - | - | - |
| 0.62035 | 1 | 3.00419 | - | - | - |
| 0.62035 | 1 | 3.00419 | - | - | - |
| 0.62035 | 1 | 3.00419 | - | - | - |
| 0.62035 | 1 | 3.00419 | - | - | - |
| 0.62035 | 1 | 3.00419 | - | - | - |
| 0.62035 | 1 | 3.00419 | - | - | - |
| 0.62035 | 1 | 3.00419 | - | - | - |
| 0.62035 | 1 | 3.00419 | - | - | - |
| 0.62035 | 1 | 3.00419 | - | - | - |
| 0.62035 | 1 | 3.00419 | - | - | - |
| 0.62035 | 1 | 3.00419 | - | - | - |
| 0.62035 | 1 | 3.00419 | - | - | - |
| 0.62035 | 1 | 3.00419 | - | - | - |
| 0.62035 | 1 | 3.00419 | - | - | - |
| 0.62035 | 1 | 3.00419 | - | - | - |
| 0.62035 | 1 | 3.00419 | - | - | - |
| 0.62035 | 1 | 3.00419 | - | - | - |
